# Jian-Pi-Bu-Xue-Formula Alleviates Cyclophosphamide-Induced Myelosuppression *via* Up-Regulating NRF2/HO1/NQO1 Signaling

**DOI:** 10.3389/fphar.2020.01302

**Published:** 2020-08-26

**Authors:** Qiuju Huang, Lizhi Feng, Hang Li, Liang Zheng, Xiaoxiao Qi, Ying Wang, Qian Feng, Zhongqiu Liu, Xiaohong Liu, Linlin Lu

**Affiliations:** ^1^Joint Laboratory for Translational Cancer Research of Chinese Medicine of the Ministry of Education of the People’s Republic of China, International Institute for Translational Chinese Medicine, Guangzhou University of Chinese Medicine, Guangzhou, China; ^2^School of Basic Medical Sciences, Guangzhou University of Chinese Medicine, Guangzhou, China; ^3^Department of Respiratory Medicine, The First Affiliated Hospital of Guangzhou University of Chinese Medicine, Guangzhou, China

**Keywords:** Jian-pi-bu-xue-formula, cyclophosphamide, complementary therapy, myelosuppression, NRF2

## Abstract

Jian-pi-bu-xue-formula (JPBXF), a TCM formula composed of twelve Chinese medicinal herbs, has been used in clinic to ease patients’ state of weakness and fatigue especially after receiving anti-tumor chemotherapy in China. The lack of the phytochemical characterization, detail therapeutic evaluation and mechanism of JPBXF remains the main limitation for its spreading. In this study, we systematically evaluated the effectiveness and underline mechanism of JPBXF on cyclophosphamide (CTX)-induced myelosuppression and identified the main constituents of JPBXF aqueous extract. JPBXF treatments reversed CTX-induced myelosuppression through increasing the number of haematopoietic stem cells (HSCs) and expression of C-kit in bone marrow cells. Simultaneously, JPBXF treatments alleviated CTX-induced blood cells reduction by increasing numbers of RBCs and WBCs and levels of GM-CSF, TPO and EPO in plasma. JPBXF treatments reduced CTX-induced immunosuppression by increasing expressions of CD3, CD4, and CD8a in PBMCs, and recovering structure damages of thymus and spleen. Moreover, JPBXF notably increased the expression of NRF2 compared with CTX group, and subsequently up-regulated HO1 and NQO1 both in mRNA and protein levels. In addition, eighteen compounds were recognized from JPBXF aqueous extract and the potential targets of the identified compounds were predicted. Overall, JPBXF can greatly reverse CTX-induced myelosuppression in C57BL/6 mice, especially in improving the blood and immune function through activating NRF2/HO1/NQO1 signaling pathway, which provides a reliable reference for JPBXF application in clinical. By recognizing eighteen compounds in JPBXF aqueous extract and predicting the underline mechanisms of the identified compounds, our study would provide theoretical guidance for further research of JPBXF.

**Graphical Abstract:**
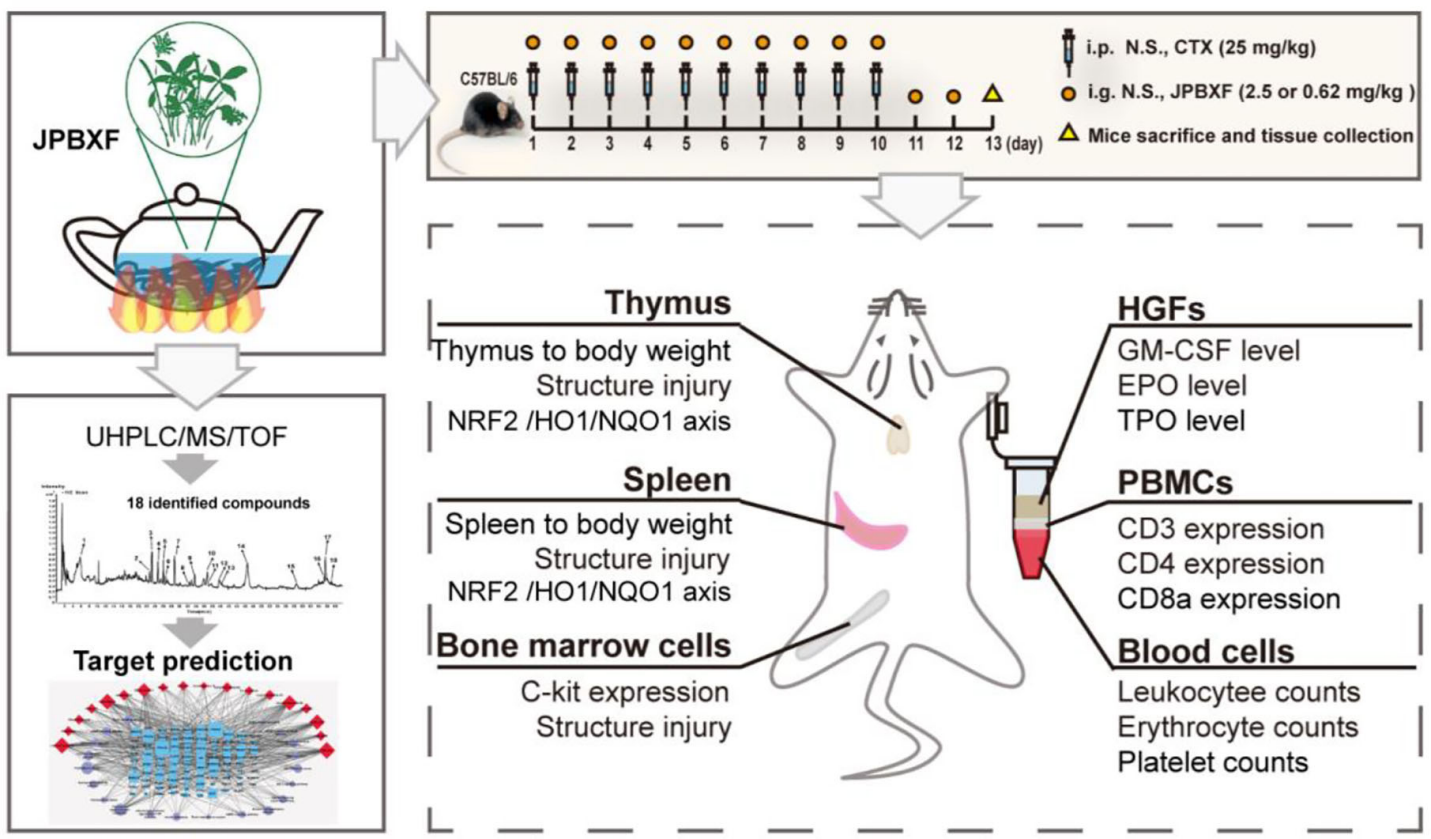


## Introduction

Cancer, an aggressive malignancy, approximately causes 4,285,033 new cases and 2,865,147 cancer-related deaths annually in China (Bray et al., 2018). The conventional therapies for cancer treatment include chemotherapy, radiotherapy and surgery. Among these treatments, chemotherapy acts as a practical method and was widely used to kill cancer cells by impeding their growth and reproduction (Potti et al., 2011). However, most of chemotherapeutic agents pose many side effects including myelosuppression, immunosuppression, gastrointestinal reaction and hepatic or renal toxicity, that greatly threaten patients’ quality of life, reduce treatments’ efficiency, and furthermore lead to termination of ongoing useful therapy (Tachi et al., 2015). For instance, cyclophosphamide (CTX), a common anti-cancer chemotherapeutic agent, is often used alone or in combination with platinum and paclitaxel as standard first-line therapy (Handolias et al., 2016). Myelosuppression, the most common side effect of CTX characterized by decrease of stem and progenitor cells in bone marrow, often arises complications such as neutropenia, anemia and thrombocytopenia (Carey, 2003; Neboh and Ufelle, 2015). It has proof that NRF2 deficiency deteriorated CTX-induced myelosuppression, while activation of NRF2 mitigates ionizing CTX -induced myelosuppression (Que et al., 2016). The up-regulation of HO1 and NQO1 could resist to CTX-induced bone marrow suppression, oxidative stress, inflammation and apoptosis (Chen et al., 2014; HAS et al., 2019). Therefore, methods, which could prevent or eliminate chemotherapy-induced side effects through regulating NRF2 pathway, have great significance for patients struggling against cancer.

Complementary medicine, forms of treatment that used along with standard medical treatments, is common among cancer patients to help lessen some side effects of cancer treatment (Hubner et al., 2015). In clinical, hematopoietic growth factors (HGFs) including granulocyte colony-stimulating factor (G-CSF) and granulocyte macrophage colony-stimulating factor (GM-CSF), are effective complementary medicines for therapy to alleviate chemotherapy-induced myelosuppression (Miller and Steinbach, 2014). Unfortunately, G-CSF and GM-CSF can also accelerate growth of cancer cells (Gutschalk et al., 2006). Therefore, new approaches on finding more effective and safe drugs for preventing and treating chemotherapy-induced myelosuppression are necessary. Traditional Chinese medicine (TCM) complementary treatment has been reported to alleviate chemotherapy induced side effects and improve quality of life (Zhang et al., 2007; Konkimalla and Efferth, 2008). For instance, *Ipomoea obscura* (L.) Ker Gawl. extract could reduce CTX-induced myelosuppression, improve white blood cell counts and recover bone marrow cellularity (Hamsa and Kuttan, 2010). Sip-jeon-dea-bo-tang, a traditional herbal medicine, has been reported to prevent the cisplatin-induced reduction of food intake and weight loss (Woo et al., 2016). Considering the limitations of hematopoietic growth factors, finding effective TCMs on alleviating chemotherapy-induced myelosuppression is helpful and meaningful.

Jian-pi-bu-xue-formula (JPBXF), a TCM consisted of twelve Chinese medicinal herbs, is consumed in clinical for strengthening spleen and fortifying the blood in China for decades. According to basic theories of Chinese medicine, JPBXF is a combined formula based on Si-Jun-Zi-Tang, Si-Wu-Tang and Dang-Gui-Bu-Xue-Tang without unnecessary drugs, aiming to improve the hematopoiesis effects and make it gently to patients with chemotherapy. JPBXF is composed of monarch drugs *Hedysarum multijugum Maxim* (Huang-Qi) and *Codonopsis pilosulae* (Franch.) Nannf. (*Codonopsis pilosulae* Radix, Dang-Shen), minister drugs including *Ficus simplicissima* Lour. syn. Ficus hirta Vahl (Wu-zhi-mao-tao), *Atractylodes macrocephala Koidz*. (Bai-Zhu)*, Angelica sinensis (Oliv.) Diels* (Dang-Gui), and *Rehmannia glutinosa* (Gaertn.) DC. (Shu-Di-Huang), assistant drugs including *Pinellia ternata* (Thunb.) Makino syn. *Arum ternatum* Thunb. (Ban-Xia), *Citrus × aurantium L*. syn. *Citrus reticulata* Blanco (Chen-Pi), *Pogostemon cablin (Blanco) Benth*. (Huo-Xiang) and *Zingiber officinale Roscoe* (Sheng-Jiang), and guide drugs *Ziziphus jujuba Mill* (Da-Zao) and *Glycyrrhiza uralensis Fisch* (Gan-Cao). In JPBXF, *H. multijugum, C. pilosula* and *R. glutinosa* had been reported to enhance immunity *in vivo* after CTX t*reatment* (Cohen et al., 2002; Wang et al., 2012). *A. sinensis* has great impacts on promoting hematopoiesis (Gong et al., 2016). *Z. jujuba, Z. officinale, A. ternatum, P. cablin, C. aurantium, A. macrocephala* and *G. uralensis* are often used in formulas for antiemetic during chemotherapy (Cohen et al., 2002). Our previous study also showed that JPBXF could improve hematopoiesis of chemotherapy mice by affecting the expression levels of GM-CSF, TPO, and EPO (Feng and Liu, 2015). Although JPBXF has been used as a formula in clinical for decades, the lack of detail therapeutic evaluation and underline mechanism of JPBXF remains the main limitation for its spreading. Furthermore, the phytochemical characterization of JPBXF is still unclear.

In our study, a CTX-induced myelosuppression animal model was established to assess the effectiveness and safety of JPBXF in alleviating chemotherapy-induced myelosuppression. Changes in thymus, spleen, bone marrow, blood cell counts, expressions of T-cell surface markers such as CD3, CD4, and CD8a in peripheral blood mononuclear cells (PBMCs), expression of C-kit in bone marrow cells, as well as levels of GM-CSF, TPO, and EPO in plasma in the mice were systematically evaluated. Meanwhile, effects of JPBXF on NRF2, HO1, and NQO1 expressions in thymus and spleen were also detected. Furthermore, main chemical constituents of JPBXF aqueous extract were identified using UHPLC/MS/TOF and the underline mechanisms of the identified compounds were predicted using bioinformatics analysis.

## Material and Methods

### Reagents

Cyclophosphamide (Pude Pharmaceutical Co., Datong, Shanxi, China) was purchased from the First Affiliated Hospital of Guangzhou University of Chinese Medicine. The anti-CD3 and anti-CD4 antibodies, goat anti-rabbit and anti-mouse secondary antibody horseradish peroxidase conjugate (IgG antibodies) were purchased from Abcam (Cambridge, Massachusett, UK). The anti-CD8a antibody and anti-C-kit receptor antibody were purchased from Santa Cruz Biotechnology (Santa Cruz, California, USA). GM-CSF, TPO, and EPO Elisa kits were purchased from R&D Systems (Minneapolis, MN., USA). All other reagents were from a standard source and were analytical pure grade.

### Plant Materials and JPBXF Preparation

*F. simplicissima* (30 g), *H. multijugum* (30 g), *C. pilosula* (20 g), *A. macrocephala* (15 g), *A. sinensis* (6 g), *R. glutinosa* (10 g), *A. ternatum* (10 g), *C. aurantium* (10 g), *P. cablin* (10 g), *Z. officinale* (6 g), *Z. jujuba* (10 g) and *G. uralensis* (10 g) were all purchased from the First Affiliated Hospital of Guangzhou University of Traditional Chinese Medicine (Guangdong, China) and identified by Institute of Chinese Materia Medica, China Academy of Chinese Medical Sciences (Beijing, China). A voucher specimen was deposited at the Laboratory of International Institute for Translational Chinese Medicine, Guangzhou University of Chinese Medicine (Guangzhou, China). The Chinese herbal mixture was immersed in a 8-fold amount of distilled water for 30 min and gently boiled for 30 min. The extract was collected. The herb residues were boiled again with a 6-fold amount of distilled water for 1 h, and the extract was collected again. Following, the collection extract were combined, filtered with three layers of gauze, and evaporated in 60 °C. After cooling at room temperature, liquid was spared and stored at 4 °C.

### JPBXF Chemical Components Analysis

Characterization of main chemical components in JPBXF was detected by UHPLC/MS/TOF (Agilent Technologies 6540). Chromatographic separation was achieved on a Zorbax C18 column (100 × 3.0 mm^2^, 1.8 μm) (Agilent Technologies), with column temperature maintained at 25°C. The mobile phases consisted of water (A) and acetonitrile (B) using a gradient elution. The flow rate was 0.3 ml/min. The mass spectrometer was operated in negative ion mode. The full scan setting parameters are as follows: capillary voltage, 2.5 kV; nozzle voltage, 1,000 V; gas temperature, 290°C; sheath temperature, 340°C; sheath gas flow, 11 L/min; and gas flow, 10 L/min; fragmentation voltage: 135 V. Self-building database containing mass spectrometry information of reported compounds from each herb was used for compounds matching.

### Animals

Male C57BL/6 mice (4–6 weeks, 18–22 g) were purchased from the Laboratory Animal Center of Guangzhou University of Chinese Medicine (Guangzhou, China; License: SCXK, Guangdong, 2018-0034). The mice were kept in the animal facility in the SPF animal laboratory (License number: SYXK (GZ) 2019-0144) at International Institute for Translational Chinese Medicine, Guangzhou University of Chinese Medicine (Guangzhou, China). The animal experiments were approved by the International Institute for Translational Chinese Medicine Animal Care and Use Committee, Guangzhou University of Chinese Medicine (Guangzhou, China). The mice randomly divided into five groups: control, CTX, GM-CSF, high- and low-dose JPBXF. Control group (n = 6) received treatments with 0.9% physiologic saline (i.p.) once a day for 10 days and intragastric administrations with 0.9% physiologic saline once a day for 12 days. CTX, GM-CSF, JPBXF groups (each with n = 6) received administrations with CTX (25 mg/kg, i.p.) once a day for 10 days and intragastric administrations with 0.9% physiologic saline, GM-CSF (5 μg/kg), high- and low-dose of JPBXF (2.5 g/mL and 0.62 g/mL) once a day for 12 days, respectively. The body weights of the mice were recorded every other day. Finally, the mice were sacrificed and the organs (including spleen and thymus) of each mouse were isolated and weighed.

### Peripheral Blood Cell Count

Before the mice were sacrificed, the blood samples (200 µL of each mouse) were collected in heparin-treated tubes by and then stored at 4°C. After all samples collection, the numbers of leukocyte, platelet, and erythrocyte were counted using SYSMEX XT-1800I (Kobe, Japan).

### Isolation of Plasma and PBMCs

After the mice were sacrificed, the whole blood of each mouse was collected in heparin-treated tubes and then centrifuged at 3,000 rpm for 10 min. The plasma samples were harvested, and stored at -80 °C. The remaining precipitation was resuspended in PBS (v/v, 1:1), and loaded into the tubes filled with Ficoll (GE Healthcare, Chicago, USA). After centrifuged at 400 g for 30 min, PBMCs were isolated, and rinsed twice with PBS.

### ELISA Assay

Levels of GM-CSF, TPO, and EPO in plasma were measured by enzyme-linked immunosorbent assay (ELISA), using Quantikine ELISA kits (R&D Systems, Minneapolis, MN, USA), according to the manufacturer’s instructions. Results were expressed as ng/L of plasma.

### Flow Cytometry

After isolated, PBMCs were treated according our previous study (Feng et al., 2016). After incubated with anti-CD3 antibody (1:200), anti-CD4 antibody (1:200), anti-CD8a antibody (1:50), and secondary antibodies (1:200), the PBMCs samples were detected using Flow cytometry (BD Biosciences, San Diego, CA, USA).

### H&E Staining

Tissues such as thymus, spleens, and femurs were treated and observed according our previous study (Feng et al., 2016). Briefly, after fixed, embedded, and sliced, sections (4 μm) of thymus, spleens, and femurs were deparaffinized and rehydrated. Following, hematoxylin and eosin staining were performed. The slices were then observed under a light microscope (Leica DM750, Wetzlar, GER).

### Western Blot

Total proteins of thymus or spleens were extracted using RIPA lysis buffer and phenylmethanesulfonyl ﬂuoride (PMSF), and quantified using Coomassie Brilliant Blue Kit (Bio-Rad, Hercules, CA, USA). Protein samples of each tissue were separated by 10% SDS-PAGE, transferred onto PVDF membranes, and blocked with 5% BSA for 1 h. The membranes were incubated with the primary antibody of NRF2 (1:1,000) at 4 °C overnight, subsequently, with the corresponding secondary antibodies (1:5,000) at room temperature for 1 h. β-actin acted as a loading control. ECL chemiluminescence reagent was applied to detect for fluorescent signals using FluorChem E (Santa Clara, CA, USA). Protein bands were quantified using Quantity One software (Bio-Rad, Hercules, CA, USA).

### Real-Time PCR Analysis

Total RNA of thymus or spleens was isolated through the TRIzol extraction method. Then, RNA reverse transcribed into cDNA according to a reverse transcription kit (TaKaRa, Shiga, Japan). SYBR Green real-time PCR amplification and detection were then performed with an ABI 7500 system (Applied Biosystems, Foster City, USA). β-actin was regarded as house-keeping gene. Primers are as follows. HO1: 5’-TGATGGCTTCCTTGTACCATATC-3’ and 3’-AGCTCCTCAGGGAAGTAGAG- 5’; NQO1: 5’-GAGAAGAGCCCTGATTGTACTG-3’ and 3’-ACCTCCCATCCTCT CTTCTT-5’; β-actin: 5’-CTGTCCCTGTATGCCTCTG-3’ and 5’-ATGTCACGCAC GATTTCC-3’.

### Immunohistochemistry (IHC)

Thymus and spleens tissues were fixed in 4% paraformaldehyde, embedded in paraffin. After sliced up, the slices (4 μm) were dewaxed, hydrated, and then incubated with natrium citric (0.01 M) for antigen retrieval. Following, the slices were rinsed with PBS, and incubated with anti-HO1 and anti-NQO1 overnight at 4 °C. Following steps were performed using the immunostaining kit (BOSTER Biological Technology) based on the manufacturer’s instructions.

### Target Prediction by Network Pharmacology

The myelosuppression-related genes were obtained from the GeneCards database (https://www.genecards.org/), and the potential target genes of compounds were predicted using a Bioinformatics Analysis Tool for Molecular mechANism of Traditional Chinese Medicine (BATMAN-TCM) (http://bionet.ncpsb.org/batman-tcm/) and Swiss Target Prediction database (http://www.swisstargetprediction.ch/). Genes obtained from these three databases were analyzed using Venny 2.1 (https://bioinfogp.cnb.csic.es/tools/venny/index.html). The gene ontology (GO) and Genomes (KEGG) enrichment analyses were performed for the potential targets using the Database for Annotation, Visualization and Integrated Discovery (DAVID) (https://david.ncifcrf.gov/) and the online software Omicshare. The protein-protein interaction (PPI) among these potential targets was constructed using the STRING database (https://string-db.org/) (Du et al., 2019). The compound-target-pathway network was constructed using Cytoscape 3.7.2 (Zhang et al., 2019).

### Data Analysis

All data were expressed as mean ± standard deviation (SD). Significant differences were analyzed by one-way ANOVA followed by LSD test (for more than two groups) by SPSS. Statistical difference was considered significant at *P* < 0.05.

## Results

### Phytochemical Characterization of JPBXF

**Figure 1:**
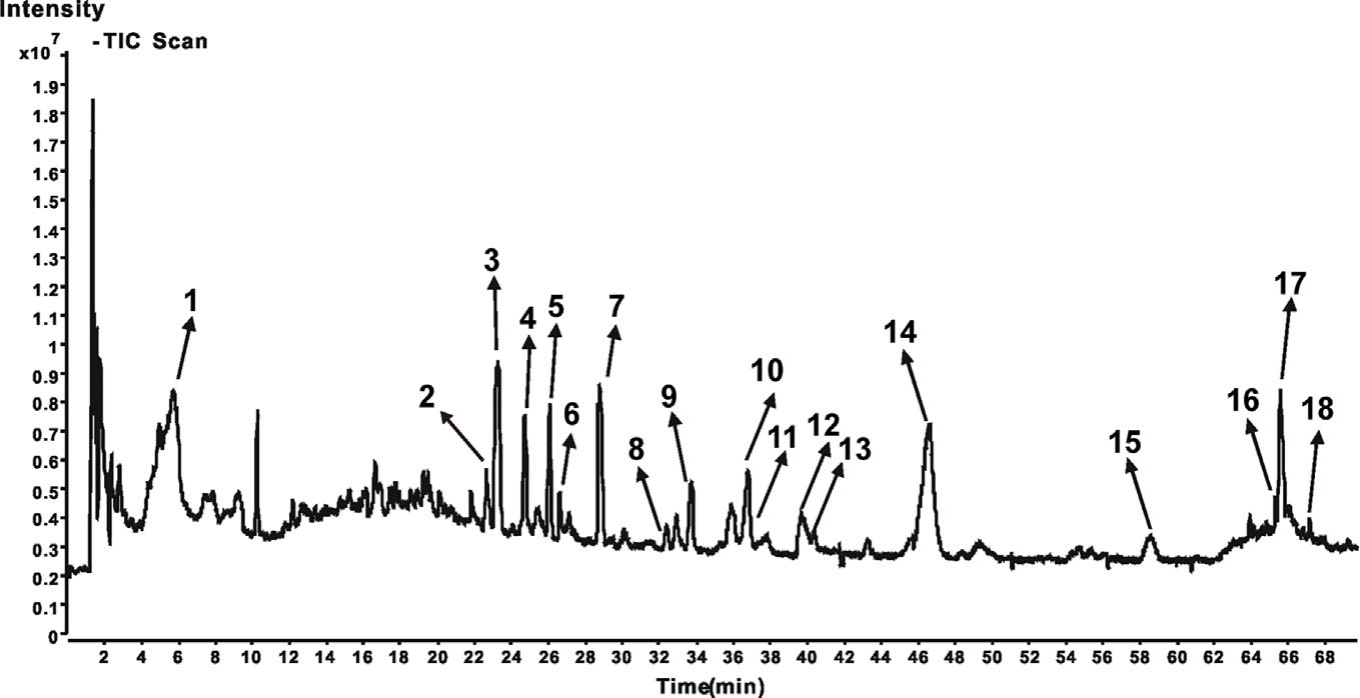
Chromatograms of UHPLC/MS/TOF of JPBXF extract.

To identify the main constituents of JPBXF aqueous extract, we analyzed the JPBXF aqueous extract using UHPLC/MS/TOF. Eighteen compounds were recognized from JPBXF aqueous extract as shown in **Table 1**. The typical scaffold backbones for the eighteen compounds are flavonoids, saponins and polyphenols. Among the eighteen compounds, there are thirteen flavonoids, including liquiritin apioside, liquiritin, acteoside, naringin, isoacteoside, hesperidin, isoliquiritin apioside, isoliquiritin, liquiritigenin, calycosin, formononetin, glycycoumarin, and licoisoflavone B. Meanwhile, licoricesaponine A3, licoricesaponine G2 and glycyrrhizic acid are three saponins. Paeonol and 4-gingerol are two polyphenols. According to UHPLC/MS/TOF, liquiritin, acteoside, naringin, hesperidin, glycyrrhizic acid and 4-gingerol were identified with high contents (**Figure 1**). Three pairs of flavonoid isomers need further identification.

**Table 1:** Compounds of aqueous extract from Jian-pi-bu-xue-formula (JPBXF) were identified.

No.	T_R_ (min)	m/z	Formula	Identification	Therotical m/z	Error (ppm)
1	5.81	165.0545	C_9_H_10_O_3_	paeonol	165.0557	7.3
2	23.21	549.1582	C_26_H_30_O_13_^a^	liquiritin apioside	549.1614	5.8
3	23.34	417.1170	C_21_H_22_O_9_^b^	liquiritin	417.1191	5.0
4	24.74	623.1952	C_29_H_36_O_15_^c^	acteoside	623.1981	4.6
5	26.07	579.1672	C_27_H_32_O_14_	naringin	579.1719	8.1
6	26.64	623.1963	C_29_H_36_O_15_^c^	isoacteoside	623.1980	2.7
7	28.74	609.1791	C_28_H_34_O_15_	hesperidin	609.1825	5.6
8	32.37	549.1589	C_26_H_30_O_13_^a^	isoliquiritin apioside	549.1614	4.5
9	33.67	417.1176	C_21_H_22_O_9_^b^	isoliquiritin	417.1191	3.6
10	36.61	983.4429	C_48_H_72_O_21_	licoricesaponine A3	983.4493	6.5
11	36.76	255.0659	C_15_H_12_O_4_	liquiritigenin	255.0663	1.6
12	39.38	283.0604	C_16_H_12_O_5_	calycosin	283.0612	2.8
13	39.91	837.3864	C_42_H_62_O_17_	licoricesaponine G2	837.3914	6.0
14	46.48	821.3915	C_42_H_62_O_16_	glycyrrhizic acid	821.3965	6.1
15	58.61	267.0655	C_16_H_12_O_4_	formononetin	267.0663	3.0
16	65.18	367.1188	C_21_H_20_O_6_	glycycoumarin	367.1187	0.3
17	65.26	265.1476	C_15_H_22_O_4_	4-gingerol	265.1445	7.9
18	67.02	351.0878	C_20_H_16_O_6_	licoisoflavone B	351.0874	1.1

^a,b,c^Three pairs of flavonoids isomers.

### Effects of JPBXF on CTX-Induced Myelosuppression

**Figure 2:**
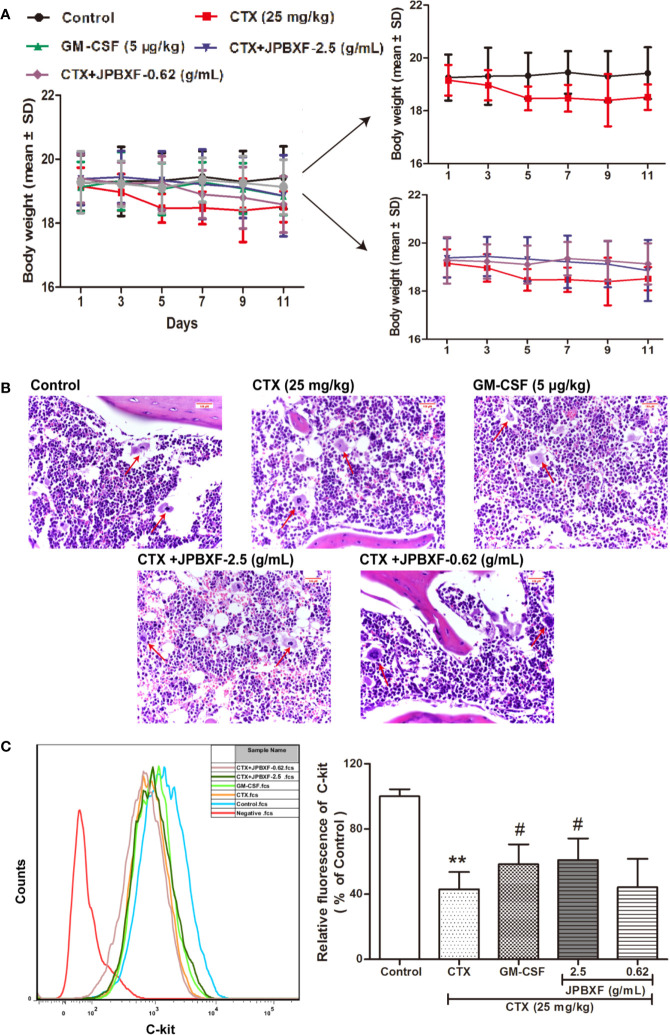
Effects of JPBXF on myelosuppression in CTX-treated mice. **(A)** The body weight curves of CTX-treated mice after JPBXF treatments. **(B)** Histopathological changes in bone marrow tissues (stained by H&E, ×400). **(C)** Expression of C-kit in bone marrow cells. Data shown are means ± SD from day 13. **P < 0.01 *vs.* Control, ^#^*P* < 0.05 *vs.* CTX.

To assess the effects of JPBXF on chemotherapy-induced myelosuppression in mice, body weight change and H&E staining of femurs were performed. Although there was no significant change in body weight among each group, CTX treatment induced a slight decrease in body-weight of mice compared with the control group (**Figure 2A**). However, after treated with JPBXF, the body-weight loss of mice induced by CTX was decreased slightly (**Figure 2A**). H&E staining results shown that CTX treatment significantly reduced the number of haematopoietic stem cells (HSCs) in femurs compared with control group. To the contrary, JPBXF treatments increased the number of HSCs compared with that of CTX treatment alone (**Figure 2B**). To further confirm whether JPBXF treatments could influence the number of HSCs, the expression of C-kit, a marker of HSCs, was detected in bone marrow cells. The results shown that compared with control, the expression of C-kit was markedly decreased by 57.02 ± 6.16% in CTX treated bone marrow cells (*P* < 0.01, **Figure 2C**). Meanwhile, 5 μg/kg of GM-CSF and 2.5 g/mL of JPBXF treatments increased the expression of C-kit by 35.68 ± 7.82% and 42.44 ± 8.71% respectively compared with that of CTX alone (*P* < 0.05, **Figure 2C**).

### JPBXF Alleviated CTX-Induced Blood Cells Reduction

**Figure 3:**
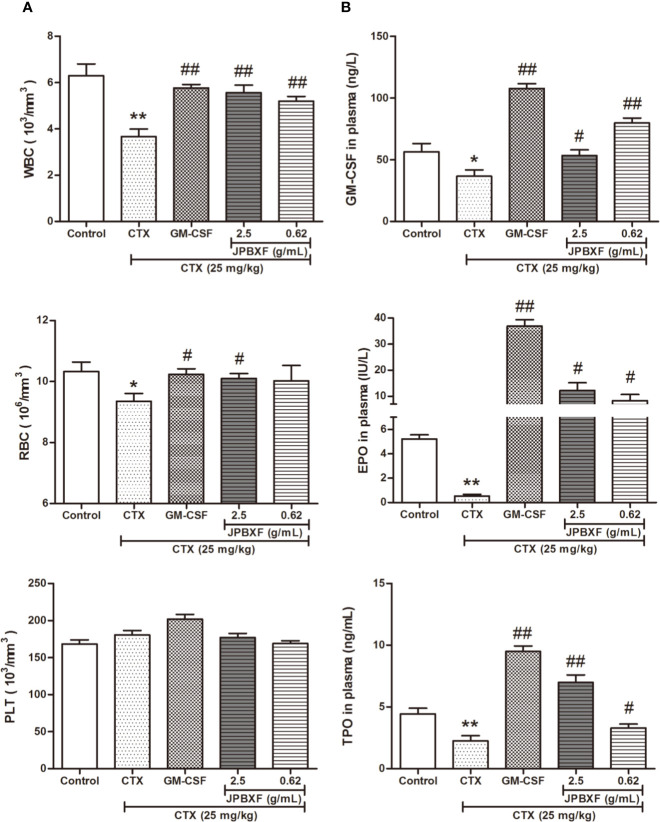
Effects of JPBXF on blood system in CTX-treated mice. **(A)** The numbers of white blood cells, red blood cells and platelets in CTX-treated mice after JPBXF treatments. **(B)** The secretion levels of GM-CSF, EPO, and TPO in plasma in CTX-treated mice after JPBXF treatments. Data shown are means ± SD from day 13. *P < 0.05, **P < 0.01 *vs.* Control, ^#^*P* < 0.05, ^##^*P* < 0.01 *vs.* CTX.

Along with myelosuppression, CTX treatments often induce blood cells reduction (Hill et al., 2011). To investigate the effects of JPBXF on blood cells, we detected the numbers of WBCs, RBCs, and PLTs and analyzed the secretion levels of hematopoietic growth factors including GM-CSF, EPO, and TPO. The whole blood analysis revealed that compared with control group, the numbers of WBCs and RBCs were significantly decreased after CTX treatments. However, 5 μg/kg of GM-CSF and 2.5 g/mL of JPBXF treatments increased the numbers of WBCs and RBCs at different degrees. Nevertheless, the number of PLTs showed a slight increase in GM-CSF group, but had few changes in CTX and JPBXF groups (**Figure 3A**). Meanwhile, ELISA assays showed that the secretion levels of GM-CSF, EPO, and TPO were significantly reduced by 35.05 ± 7.99%, 89.84 ± 2.48%, and 49.48 ± 4.79% respectively in CTX group compared with the control group. However, JPBXF treatments abrogated the suppression effect of CTX and markedly increased the secretion levels of GM-CSF, EPO, and TPO at different degrees in plasma. Compared with CTX group, 2.5 g/mL of JPBXF treatments increased the levels of GM-CSF, EPO and TPO by 2.94-, 69.84-, and 4.21-fold in plasma (**Figure 3B**).

### JPBXF Reduced CTX-Induced Immunosuppression

**Figure 4:**
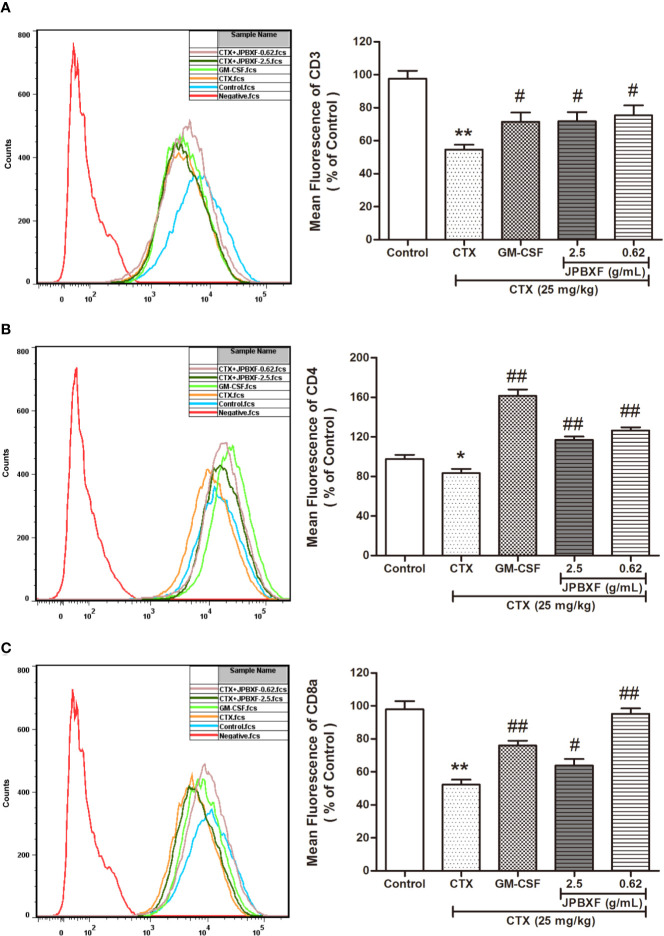
Effects of JPBXF on the PBMCs in CTX-treated mice. The expression levels of CD3 **(A)**, CD4 **(B)**, and CD8α **(C)** in peripheral blood mononuclear cells (PBMCs) of CTX-treated mice after JPBXF treatments. Data shown are means ± SD from day 13. *P < 0.05, **P < 0.01 *vs.* Control, ^#^*P* < 0.05, ^##^*P* < 0.01 *vs.* CTX.

According to the white blood cell analysis results, we found that JPBXF treatments might raise the immunity of mice suppressed by CTX. Among these white blood cells, T cell play important role in immunity. To further confirm the effect of JPBXF on T cells, we detected the expressions of T-cell surface markers, such as CD3, CD4, and CD8α in PBMCs. The results demonstrated that compared with control group, CTX treatment significantly decreased the expressions of CD3, CD4, and CD8α by 44.03 ± 2.96%, 14.63 ± 4.14%, and 46.70 ± 3.10%, respectively. However, CTX-induced expression decrease of CD3, CD4 and CD8α were recovered by JPBXF treatments (**Figure 4**). In detail, 2.5 g/mL of JPBXF treatments increased CD3, CD4, and CD8a expression levels by 31.33 ± 5.59%, 28.62 ± 3.44%, and 18.13 ± 2.24%, respectively. Meanwhile, 0.62 g/mL of JPBXF treatments increased the expressions of CD3, CD4, and CD8a by 37.99 ± 5.07%, 34.06 ± 3.09%, and 45.16 ± 3.29%, respectively.

**Figure 5:**
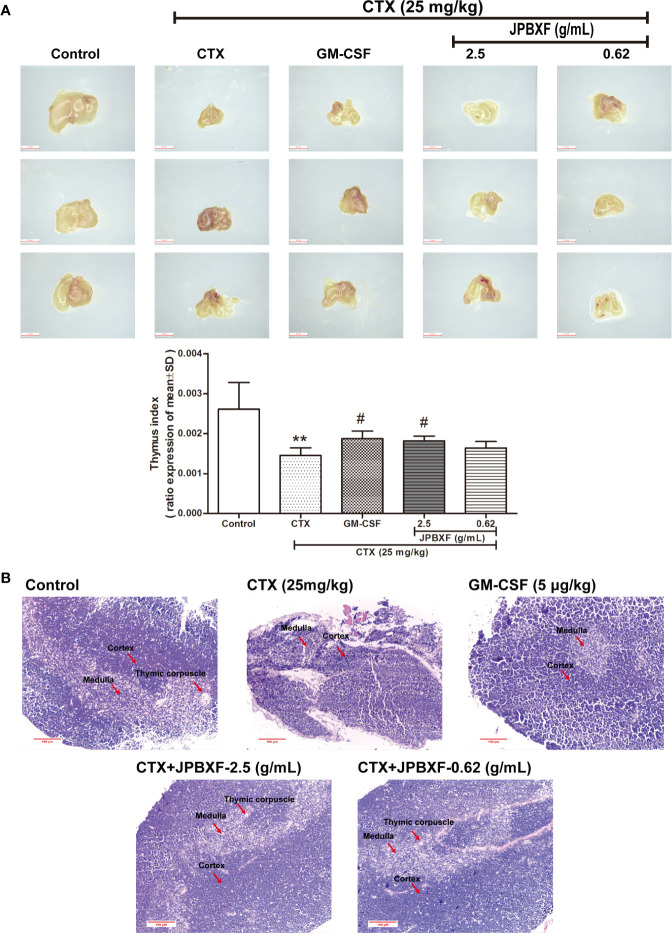
Effects of JPBXF on the thymus in CTX-treated mice. **(A)** Sizes and index of thymus in mice (scale = 3 mm). **(B)** Histopathological changes in thymus tissues (stained by H&E, ×200). Data shown are means ± SD from day 13. **P < 0.01 *vs.* Control, ^#^*P* < 0.05 *vs.* CTX.

**Figure 6:**
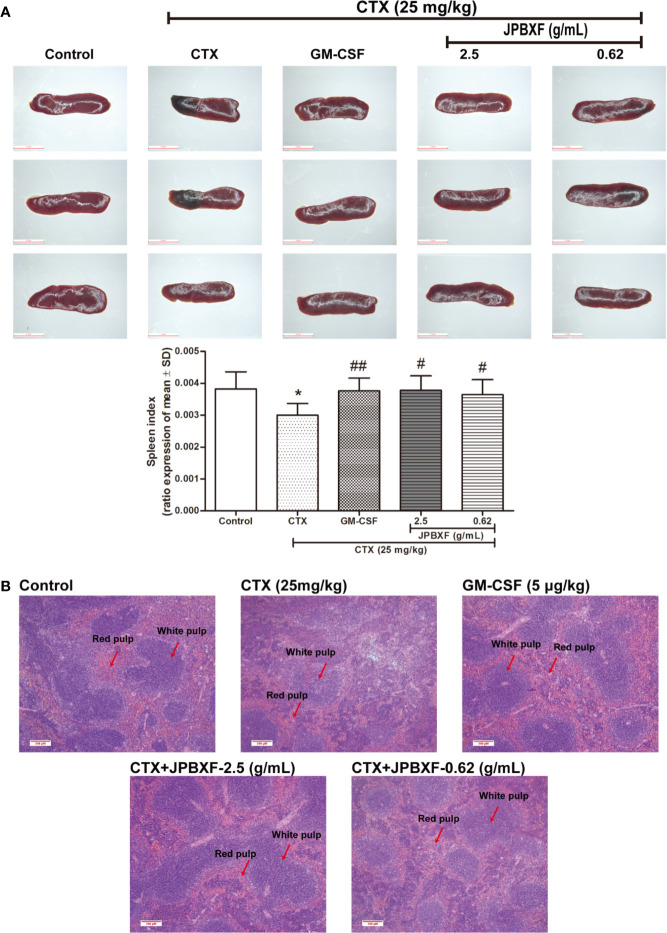
Effects of JPBXF on the spleen in CTX-treated mice. **(A)** Sizes and index of spleens in mice (scale=5 mm). **(B)** Histopathological changes in spleens tissues (stained by H&E, ×100). Data shown are means ± SD from day 13. *P < 0.05 *vs.* Control, ^#^*P* < 0.05, ^##^*P* < 0.01 *vs.* CTX.

Thymus and spleen are two main immune organs that play important roles in cellular and humoral immunity. To assess the effects of JPBXF on cellular and humoral immunity in mice, size comparison and H&E staining of thymus and spleen were further conducted. The pictures of thymus directly showed that CTX treatment induced a size reduce of thymus compared with that of control, however, JPBXF treatments increased the size of thymus. Compared with the control group, thymus index (ratios of thymus to body-weight) in CTX group decreased markedly. Meanwhile, thymus index in GM-CSF group and high-dose JPBXF group were significantly increased compared with CTX group; however, thymus index in the low-dose JPBXF group had no significant change (**Figure 5A**). H&E staining results showed that the tissue structures of thymus, including cortex, medulla, and thymic corpuscle, were clear observed in control group, and destroyed in CTX group. However, the structure damages of thymus by CTX were significantly recovered by JPBXF treatments (**Figure 5B**). The same as thymus, the size of spleen was reduced by CTX and increased by JPBXF. Spleen index (ratios of spleen to body-weight) in CTX group was decreased markedly compared with that of control group, meanwhile, increased in GM-CSF and JPBXF groups compared with CTX group (**Figure 6A**). In addition, white pulp atrophy, hemorrhage and necrosis were observed in spleen after CTX treatment. However, these damages were reduced in GM-CSF and JPBXF groups, especially in 2.5 g/mL JPBXF group (**Figure 6**).

### JPBXF Alleviated CTX-Induced Myelosuppression Through Activating NRF2/HO1/NQO1 Signaling Pathway

**Figure 7:**
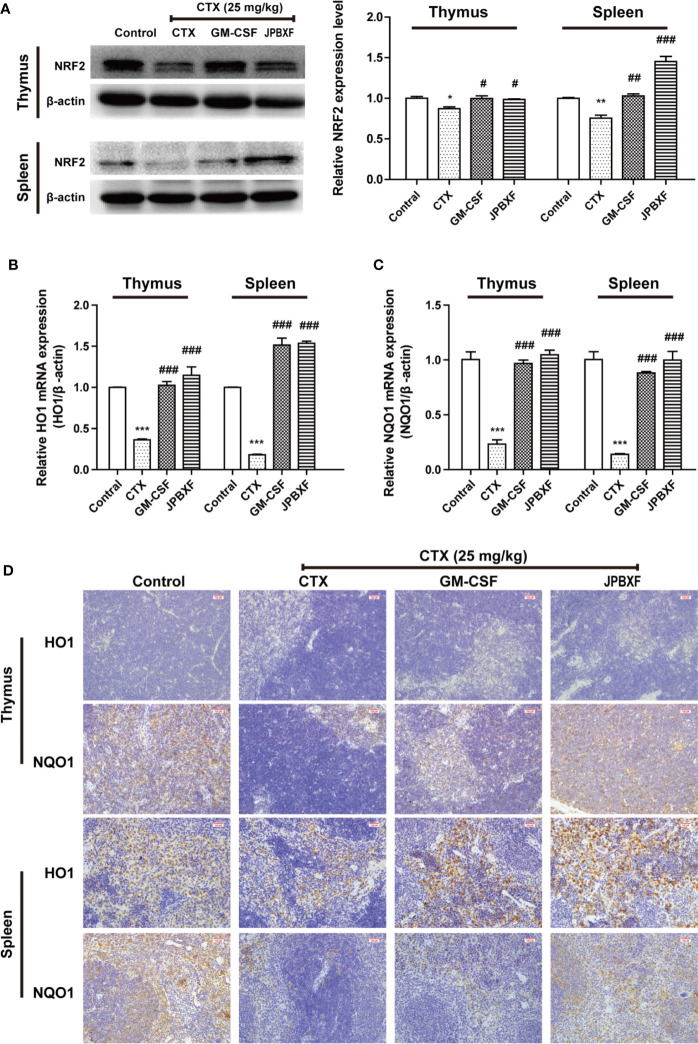
JPBXF activated NRF2/HO1/NQO1 pathway. **(A)** Protein expressions of NRF2 in thymus or spleen tissues after treatments as design. mRNA expression levels of HO1**(B)** and NQO1 **(C)** were detected using real-time PCR analysis. **(D)** Images of HO1 and NQO1 protein expressions in thymus or spleen tissues were presented by immunohistochemistry. Data shown are means ± SD from day 13. *P < 0.05, **P < 0.01, ***P < 0.001 *vs.* Control, ^#^*P* < 0.05, ^##^*P* < 0.01, ^###^*P* < 0.001 *vs.* CTX.

NRF2, a pivotal transcription factor, is involved in regulating redox homeostasis, drug metabolism, responses to oxidative and electrophilic stress and so on (Hayes and Dinkova-Kostova, 2014). NRF2 deficiency deteriorated CTX-induced myelosuppression, while activation of NRF2 mitigated CTX-induced myelosuppression (Que et al., 2016). Herein, to further determine the underlying mechanism of JPBXF in alleviating CTX-induced myelosuppression, NRF2 expressions in thymus or spleen were evaluated. Our results showed that the expressions of NRF2 were significantly decreased by CTX both in thymus and spleen compared with control group, meanwhile, notably increased by GM-CSF and JPBXF treatments compared with CTX group, suggesting that JPBXF could mitigate CTX-induced myelosuppression through activating of NRF2 (**Figure 7A**). The up-regulation of HO1 and NQO1 could resist to CTX-induced bone marrow suppression, oxidative stress, inflammation and apoptosis (Chen et al., 2014; HAS et al., 2019), and we found that JPBXF reversed CTX-induced HO1 and NQO1 suppressions both in mRNA and protein levels (**Figures 7B–D**), indicating that JPBXF alleviated CTX-induced myelosuppression through activating NRF2/HO1/NQO1 signaling pathway.

### Target Prediction of Eighteen Compounds on Alleviating Myelosuppression by Network Pharmacology

**Figure 8:**
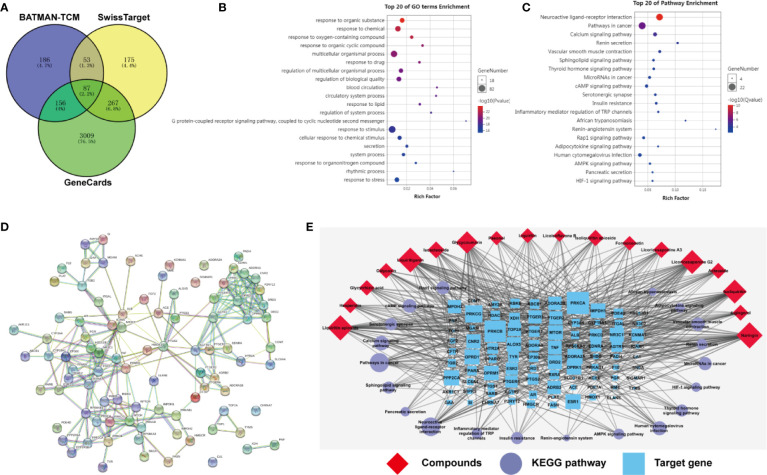
Target prediction of eighteen compounds on alleviating myelosuppression. **(A)** The 87 potential target genes of eighteen compounds on myelosuppression were selected using Venny. **(B)** Gene ontology (GO) enrichment analysis of 87 selected genes performed by Database for Annotation, Visualization and Integrated Discovery (DAVID) and visualized by Omicshare. **(C)** KEGG enrichment analysis of 87 selected genes performed by DAVID and visualized by Omicshare. **(D)** The protein-protein interaction (PPI) network of 87 selected genes constructed by the STRING database. **(E)** The compound-target-pathway network was constructed and visualized by Cytoscape.

We queried 3,519 genes related to myelosuppression using GeneCards database, 482 predicted target genes of compounds using BATMAN-TCM database and 582 predicted target genes of compounds using Swiss Target Prediction database. 87 of the potential targets yielded from these three database were selected for GO and KEGG analyses (**Figure 8A**). P < 0.05 was set as threshold criteria to identify the functional gene ontology and pathway, and Top 20 ontology and pathway were showed in **Figures 8B, C**. GO enrichment analysis indicated that the 87 potential targets were primarily associated with the “response to organic substance,” “response to chemical,” “multicellular organismal process,” “regulation of multicellular organismal process,” and “regulation of biological quality” terms. KEGG enrichment analysis revealed that the 87 potential targets were significantly enriched in the “Neuroactive ligand-receptor interaction,” “Pathways in cancer,” “Calcium signaling pathway” “cAMP signaling pathway” and “Vascular smooth muscle contraction” terms. The PPI network identified 14 key genes for the compounds, including AGTR2, PTGS2, MTOR, DRD2, EP300, OPRM1, AGTR1, DRD3, OPRD1, OPRK1, PPARG, PTGER3, RXRA, and TNF (**Figure 8D**). The three-level network consisted of 125 nodes (18 compounds, 87 genes and 20 pathways) and 552 edges. Among the 18 compounds, naringin showed the broadest effect on the target genes (38 genes). Liquiritin apioside, liquiritigenin, licoricesaponine G2, and isoliquiritin also affected 36, 35, 33, and 33 genes, respectively. Among the 87 genes, PRKCA, PRKCB and IMPDH1 were influenced by 16, 13, and 12 compounds, respectively. OPRD1, PPP2CA, ESR1, OPRM1, OPRK1, and ALOX5 were affected by 11 compounds. CNR2 was affected by 10 compounds. IMPDH2 was influenced by 9 compounds. DRD2, NR3C1 and TOP2A were affected by 8 compounds. While three targets were influenced by 7 compounds, one target was influenced by 6 compounds, ten targets were affected by 5 compounds, nine targets were affected by 4 compounds, twenty three targets were affected by 3 compounds, and the rest twenty seven were affected by only one compound.

## Discussion

TCMs are widely used in Asian countries, especially China, Japan and Korea. With the development of medicine, Chinese medicine is gradually accepted by western countries (Dobos and Tao, 2011). Given the ability of alleviating chemotherapy-induced side effects, TCMs complementary treatment is widely adopted for patients suffering from cancers (Woo et al., 2016). To be an empirical TCM formula, JPBXF has been used to ease chemotherapy-induced weakness and fatigue for cancer patients in clinic in China for decades. However, the phytochemical characterization, detail therapeutic evaluation and underline mechanism of JPBXF remain unclear.

Our study directly provides evidence that JPBXF treatments could alleviate CTX-induced myelosuppression in C57BL/6 mice as following aspects: (**i)** JPBXF treatments increased the number of HSCs; (**ii)** JPBXF treatments recovered CTX-induced blood cells reduction; (**iii)** JPBXF treatments reduced CTX-induced immunosuppression. Cancer patients who receive CTX-chemotherapy, often suffered side effects including myelosuppression, weight loss, asthenia, and so on (Strati et al., 2013; Que et al., 2016). In our study, JPBXF treatments could increase the number of HSCs and the expression of HSC marker C-kit (**Figure 2**), illustrating that JPBXF could abrogate the myelosuppression induced by CTX. On the other hand, CTX treatment could significantly reduce the number of WBCs and RBCs and the secretion levels of GM-CSF, EPO and TPO in mice, which consistent with the previous reports (Botnick et al., 1981; Jantunen et al., 2003). Expectedly, JPBXF treatments increased the number of RBCs and WBCs, and the secretion levels of EPO and GM-CSF at different degrees (**Figure 3**). Except for increasing blood function, JPBXF also showed great ability on reducing CTX-induced immunosuppression in C57BL/6 mice. It is reported that CTX treatment could induce immunosuppression and inhibit the generation and function of T cells (Traverso et al., 2012; Salva et al., 2014). CD3, CD4, and CD8a are the most abundant immunity T cell in the PBMCs (Sedgmen et al., 2013). We found that the expressions of CD3, CD4, and CD8a in PBMCs were decreased by CTX, while JPBXF abrogated the decrease expressions of CD3, CD4 and CD8a (**Figure 4**). Furthermore, thymus and spleen are two main immune organs that play important roles in cellular and humoral immunity. Thymus and spleen functions can be inhibited by CTX treatment (Zhao et al., 2009). In the present study, we found that thymic atrophy and splenatrophy were obvious after CTX treatment. To the contrary, thymus and spleen index increased after JPBXF treatments (**Figures 5A** and **6A**). In addition, CTX-induced structure damages, including cortex, medulla and thymic corpuscle in thymus and white pulp atrophy, hemorrhage and necrosis in spleen, were recovered by JPBXF treatments (**Figures 5B** and **6B**).

Our data also revealed that JPBXF alleviated CTX-induced myelosuppression through activating NRF2/HO1/NQO1 signaling pathway. It is reported that NRF2 deficiency deteriorated CTX-induced myelosuppression, while activation of NRF2 mitigates CTX -induced myelosuppression (Que et al., 2016). The up-regulation of HO1 or NQO1 could resist to CTX-induced bone marrow suppression, oxidative stress, inflammation and apoptosis (Chen et al., 2014; HAS et al., 2019). We observed that JPBXF treatment notably reversed CTX-induced NRF2 suppressions both in thymus and spleen, subsequently, recovered the expression of HO1 and NQO1 both in mRNA and protein levels (**Figure 7**), indicating that JPBXF alleviated CTX-induced myelosuppression through activating NRF2/HO1/NQO1 signaling pathway.

In our study, JPBXF aqueous extract showed great ability in alleviating CTX-induced myelosuppression in C57BL/6 mice. However, the main chemical constituents in JPBXF aqueous extract and the underline mechanisms of the main compounds on alleviating CTX-induced myelosuppression are still unclear. Herein, we identified main chemical constituents of JPBXF aqueous extract using UHPLC/MS/TOF and confirmed that the main constituents in JPBXF extracts are paeonol, liquiritin apioside, liquiritin, acteoside, naringin, isoacteoside, hesperidin, isoliquiritin apioside, isoliquiritin, licoricesaponine A3, liquiritigenin, calycosin, licoricesaponine G2, glycyrrhizic acid, formononetin, glycycoumarin, 4-gingerol, and licoisoflavone B (**Figure 1** and **Table 1**). We also try to explain the underline mechanism by which 18 compounds on alleviating CTX-induced myelosuppression using bioinformatics analysis and found that 87 potential targets were primarily associated with the “response to organic substance,” “response to chemical,” “multicellular organismal process,” “regulation of multicellular organismal process,” and “regulation of biological quality” terms by targeting multi-protein network, such as “Neuroactive ligand-receptor interaction,” “Pathways in cancer,” “Calcium signaling pathway” “cAMP signaling pathway,” and “Vascular smooth muscle contraction”. Combining with the PPI network and the three-level network results, 24 identified genes, including AGTR2, PTGS2, MTOR, DRD2, EP300, OPRM1, AGTR1, DRD3, OPRD1, OPRK1, PPARG, PTGER3, RXRA, TNF, PRKCA, PRKCB, IMPDH1, PPP2CA, ESR1, ALOX5, CNR2, IMPDH2, NR3C1, and TOP2A (**Figure 8**), might be the key targets of 18 compounds in alleviating CTX-induced myelosuppression. To confirm these prediction results, more researches will be conducted in the future.

In conclusion, JPBXF can greatly reverse CTX-induced myelosuppression in C57BL/6 mice, especially in improving the blood and immune function through activating NRF2/HO1/NQO1 signaling pathway, which provides a reliable reference for JPBXF application in clinical. By recognizing 18 compounds in JPBXF aqueous extract and predicting the underline mechanism, our study would provide theoretical guidance for further research of JPBXF.

## Data Availability Statement

The raw data supporting the conclusions of this article will be made available by the authors, without undue reservation, to any qualified researcher.

## Ethics Statement

The animal study was reviewed and approved by International Institute for Translational Chinese Medicine Animal Care and Use Committee, Guangzhou University of Chinese Medicine.

## Author Contributions

LL and XL designed the research. QH, LF, HL, and LZ performed the study. QH and LF analyzed the data and wrote the manuscript. ZL and QF revised the manuscript. XQ and YW provided technical support. All authors contributed to the article and approved the submitted version.

## Funding

This work was supported by the projects of the National Natural Science Foundation of China [81720108033, 81930114, and 81874367], the Natural Science Foundation of Guangdong Province [2018B030322011], and the Natural Science Funds for Distinguished Young Scholar of Guangdong Province [2017A030306033].

## Conflict of Interest

The authors declare that the research was conducted in the absence of any commercial or financial relationships that could be construed as a potential conflict of interest.

## Abbreviations

BATMAN-TCM, Bioinformatics Analysis Tool for Molecular mechANism of Traditional Chinese Medicine; CTX, cyclophosphamide; DAVID, Database for Annotation, Visualization and Integrated Discovery; ELISA, enzyme-linked immunosorbent assay; EPO, erythropoietin; G-CSF, granulocyte colony-stimulating factor; GM-CSF, granulocyte macrophage colony-stimulating factor; JPBXF, Jian-pi-bu-xue-formula; PBMC, peripheral blood mononuclear cell; PPI, protein-protein interaction; TCM, traditional Chinese medicine; TPO, thrombopoietin.
